# Clinical value of integrated‐signature miRNAs in esophageal cancer

**DOI:** 10.1002/cam4.1129

**Published:** 2017-07-14

**Authors:** Heng‐Chao Zhang, Kai‐Fu Tang

**Affiliations:** ^1^ Institute of Translational Medicine The First Affiliated Hospital of Wenzhou Medical University Wenzhou 325015 Zhejiang China; ^2^ Digestive Cancer Center The First Affiliated Hospital of Wenzhou Medical University Wenzhou 325015 Zhejiang China

**Keywords:** Enrichment analysis, esophageal cancer, prognosis

## Abstract

MicroRNAs (miRNAs) are crucial regulators of gene expression in tumorigenesis and are of great interest to researchers, but miRNA profiles are often inconsistent between studies. The aim of this study was to confirm candidate miRNA biomarkers for esophageal cancer from integrated‐miRNA expression profiling data and TCGA (The Cancer Genome Atlas) data in tissues. Here, we identify five significant miRNAs by a comprehensive analysis in esophageal cancer, and two of them (hsa‐miR‐100‐5p and hsa‐miR‐133b) show better prognoses with significant difference for both 3‐year and 5‐year survival. Additionally, they participate in esophageal cancer occurrence and development according to KEGG and Panther enrichment analyses. Therefore, these five miRNAs may serve as miRNA biomarkers in esophageal cancer. Analysis of differential expression for target genes of these miRNAs may also provide new therapeutic alternatives in esophageal cancer.

## Introduction

Esophageal cancers, including esophageal adenocarcinoma and esophageal squamous cell carcinoma, were the fifth highest in cancer incidence in China, accounting for 50% of all new cases with liver cancer and 49% of cancer‐related deaths worldwide according to the 2014 World Cancer Report 1. At present, the best strategy to improve the prognosis of esophageal cancer is early detection, diagnosis, and treatment. However, accurate diagnosis with an effective treatment strategy is still a challenge. Several miRNAs have been identified to play fundamental roles in the occurrence and development of cancer, and show altered expression in human various cancers 2, which motivated us to further explore the potential application of miRNAs in esophageal cancer diagnosis and therapy.

miRNA is a single‐stranded noncoding RNA consisting of 20–24 nucleotides, which functions in suppressing target gene expression by binding to complementary sequences in the 3__‐untranslated region of mRNAs, leading to degradation of mRNA and inhibition of their translation 3. Complex interactions existing among miRNAs, target genes, and phenotypes have emerged as valuable biomarkers of diagnosis and prognosis associated with various phenotypes in diseases. Since the first report of a direct link between miRNAs and human cancer, in which miR‐15 and miR‐16 were found to be absent or downregulated in B‐cell chronic lymphocytic leukemia, many studies have evaluated their biological roles and associations with disease 4. Owing to innovations in biotechnology, large datasets have emerged, providing a substantial amount of valuable miRNA data. However, miRNA profiling efforts are often inconsistent between studies because of small sample size, different technological platforms, and different methods for processing and analysis. To overcome these problems, a more advanced and powerful strategy is necessary to handle these comprehensive and complex high‐throughput data. These specific miRNA biomarkers could be applied in early diagnosis and therapy of esophageal cancer in the future.

## Methods

### Data collection and processing

We search for articles in PubMed published from 1999 to 2016 (last accessed on 20 January 2016), by means of the combination of the following key terms: ((esophageal and (cancer* or carcinoma or tumour* or tumor*)) and (microRNA* or miRNA* or miR‐*) and profil*. In total, we obtain 302 relevant articles, and 217 of them could be downloaded with full text and miRNA profiles from English‐language journals. Further screening filters out 48 papers in which the patient samples are cancer tissues and paired adjacent noncancerous tissues, and not serum samples. To maintain the strategic accuracy and standardize these data as much as possible, the fold change in each miRNA expression is regarded as rule for further screening, and then 12 articles are retained finally.

We download and extract the TCGA data including miRNA expression data, gene expression data and patients' clinical information from the TCGA Data Portal (https://tcga-data.nci.nih.gov/tcga/; last release, March 2016) 5. Gene expression profiles (Reads per kilobase per million, RPKM) and miRNA expression profiles (reads per million, RPM) are log_2_‐transformed and used for subsequent analysis. In total, 13 pairs of samples are applied to evaluate the expression levels of miRNAs by paired‐samples *t*‐test and their target genes by the DESeq2 program 6 between solid tumors and adjacent noncancerous tissues. The clinical record in 185 esophageal cancer samples is for prognosis analysis.

### Standardization of miRNA data

Frequently, a miRNA exists as various precursors in primary data. Additionally, the mature form of miRNA always plays crucial roles in regulation of target gene expression. To analyze these data with greater precision and high efficiency, a unified standardization strategy is applied such that the precursors or alias of each miRNA are converted to the corresponding mature form according to the miRBase database (http://www.mirbase.org/; last release, Release 21 in June 2014) 7.

### RRA analysis

The novel RRA method 8, based on the leave‐one‐out cross‐validation and Bonferroni correction, assigns a *P*‐value to each miRNA in the last aggregated list. The *P*‐value for each miRNA indicates how much better it is ranked compared with a null model, expecting random ordering. With the RRA method, we analyze the ranking miRNA lists based on their fold change in expression level from 12 studies to obtain meta‐signature miRNA. Here, the *P*‐value for each miRNA can indicate whether its expression in esophageal cancer tissues is statistically significant or not, compared with paired normal tissue. The RRA approach is openly available in Comprehensive R Archive Network (http://cran.r-project.org/).

### Prediction, integration, and verification of target genes

Target genes of these five miRNAs in esophageal cancer are predicted with the miRDB database (http://mirdb.org/miRDB/) 9, TargetScan database (http://www.targetscan.org/) 10, microT‐CDS database (http://www.microrna.gr/microT-CDS) 11, and RNA22 database (http://cm.jefferson.edu/rna22/) 12. Additionally, the experimentally validated target genes of these five miRNAs are searched with the DIANA‐TarBase v7.0 database (http://www.microrna.gr/tarbase) 13 and the MiRTarBase database (http://mirtarbase.mbc.nctu.edu.tw/) 14. Then, the target gene lists for each miRNA are generated based on the intersection of the four predicted miRNA target gene databases (miRDB, TargetScan, microT‐CDS, and RNA22) and the union of the two experimentally validated miRNA targeted‐gene databases (DIANA‐TarBase v7.0 and MiRTarBase). Finally, the expression levels of these target genes are tested between 13 paired esophageal cancer and adjacent noncancerous tissues from the TCGA data to obtain significantly differently expressed genes. In theory, upregulation of miRNA expression would lead to downregulation of its target genes and vice versa. Thus, the target genes are confirmed with a further filter (downregulated miRNA: *P* ≤ 0.05 and log_2_ [fold change] >0; upregulated miRNA: *P* ≤ 0.05 and log_2_ [fold change] <0).

### Enrichment analysis

To elucidate the biological function of these miRNAs, the candidate target genes are subjected to functional enrichment analyses individually with GeneCodis3 software. The GeneCodis3 (http://genecodis.cnb.csic.es/), a web‐based application for singular and modular enrichment analysis, integrates information for various types of data (functional, regulatory, and structural) by searching for frequent patterns among annotations and evaluating statistical relevance 15. This new approach, superior to DAVID, Onto‐Express, ProfCom or FATIGO+ by overcoming the lack of term–term relationships in these analyses, profiles different sides of the same information and offers a more accurate interpretation of the data.

GeneCodis3 is applied to the functional enrichment analyses (KEGG and Panther pathways) of the significant target genes for each miRNA with a hyp‐c ≤0.05. Here, the hyp‐c, the *P*‐value for the hypergeometric test with multiple hypothesis corrections, represents the significance of the association between each enrichment pathway and the input list of genes.

### Prognosis for differentially expressed miRNAs

The association between miRNA expression and survival for esophageal cancer patients is explored by separating the cases from each cohort into a group with a high expression level and another with a low expression level. The data‐driven approach 16, a novel computational method for the identification of miRNAs with a significant influence on survival and patient grouping, estimates the optimal threshold expression level for each miRNA for grouping of patients by maximizing the separation of the survival curves related to the risks of the disease. The log‐rank test, based on Kaplan–Meier plots, determines the differences among survival curves with respect to the miRNA expression levels. The univariate HR value, based on the Wald's test, determines statistical significance along with 95% CIs in the Cox proportional hazards model.

## Results

### Confirmation of five significantly differentially expressed miRNAs in esophageal cancer

**Figure 1:**
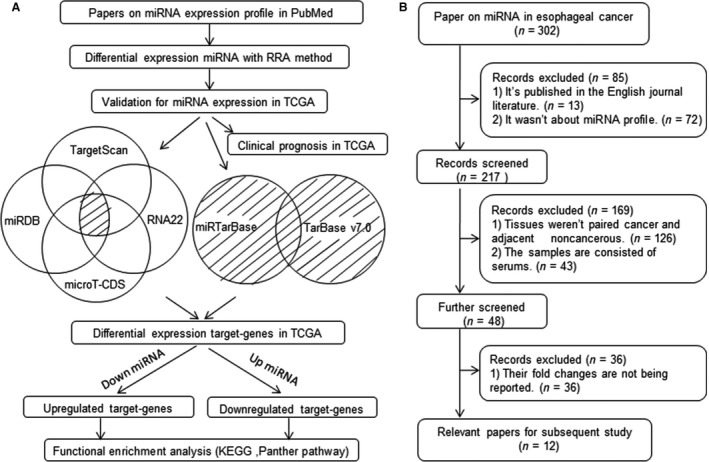
Flowchart for the study. (A) General strategy of the study. The RRA method (novel robust rank aggregation) was applied to obtaining significant miRNAs. Six databases including TargetScan, miRDB, microT‐CDS, RNA22, miRTarBase, and TarBase v7.0 were applied to obtaining the target genes of the miRNA. The dashed area in A represents the target genes we need. TCGA: The Cancer Genome Atlas. (B) Screening rules for articles on the miRNA expression profile in PubMed.

The general strategy of our study is described in detail in Figure 1A. We obtained 12 relevant articles 17, 18, 19, 20, 21, 22, 23, 24, 25, 26, 27, 28 from the 302 articles with the screening criteria (Fig. 1B; Table 1). The miRNAs data from these 12 articles were then extracted and analyzed with the novel robust rank aggregation (RRA) method 29 to identify miRNAs with statistically significant differences in expression. Overall, 217 miRNAs were upregulated by >2‐fold and 97 miRNAs were downregulated by <0.5‐fold. Among them, 14 miRNAs showed integrated significance with the RRA method, including four upregulated miRNAs and 10 downregulated miRNAs (Table 2). To validate these 14 miRNAs, their expression levels in 13 paired esophageal cancer and adjacent noncancerous tissue samples from TCGA were reanalyzed. Ultimately, five of them showed significantly different expression, including three upregulated miRNAs (hsa‐miR‐155‐5p, hsa‐miR‐21‐5p, and hsa‐miR‐223‐3p) and two downregulated miRNAs (hsa‐miR‐100‐5p and hsa‐miR‐133b), with *P* ≤ 0.05 (Table 2). Notably, previously published quantitative real‐time PCR analyses (RT‐PCR) further supported our results. Hsa‐mir‐155 was upregulated in esophageal squamous cell carcinoma tissues compared to paired adjacent noncancerous tissues 30. At least two independent studies found that hsa‐mir‐21 was significantly upregulated in tumors compared to normal tissues 31, 32. Expression of hsa‐mir‐223 was also markedly increased 33, 34. Hsa‐mir‐100 35 and hsa‐mir‐133b 36, 37 were notably downregulated in tumor tissues compared to normal tissues. Collectively, these results conclusively demonstrate that compared with adjacent noncancerous tissues, these five miRNAs show significantly different expression in esophageal cancer tissues.

**Table 1:** Characteristics of the analyzed articles

Reference	Country	Cancer type	No. of tissue samples (cancer/normal)	Technique	No. of miRNAs in show
Feber (2008)	USA	EAC	20 (10/10)	Microarray	14
Wijnhoven (2009)	Australia	EAC	14 (7/7)	Microarray	44
Fu (2013)	China	ESCC	68 (34/34)	Microarray	12
Kong (2011)	China	ESCC	10 (5/5)	Microarray	22
Hong (2010)	China	ESCC	20 (10/10)	Microarray	12
Kano (2010)	Japan	ESCC	20 (10/10)	Microarray	15
Yang (2013)	China	ESCC	6 (3/3)	Microarray	15
Fu (2013)	China	ESCC	18 (9/9)	Microarray	18
Saad (2013)	USA	EAC	68 (34/34)	Microarray	21
Liu (2013)	China	EAC	6 (3/3)	Microarray	60
Zang (2013)	China	ESCC	6 (3/3)	Microarray	65
Wu (2013)	USA	EAC	70 (35/35)	Microarray	138

EAC, esophageal adenocarcinoma; ESCC, esophageal squamous cell carcinoma.

**Table 2:** miRNA expression of esophageal cancer samples in published articles and the TCGA

miRNA	Stem‐loop	miRNA expression in article		miRNA expression in TCGA	
		Tumor versus noncancerous	*P*‐value	log_2_ (fold change)	*P*‐value
**hsa‐miR‐100‐5p**	hsa‐mir‐100	Downregulated	0.00002	−1.695	0.04018
**hsa‐miR‐133b**	hsa‐mir‐133b	Downregulated	0.02949	−2.097	0.00451
**hsa‐miR‐155‐5p**	hsa‐mir‐155	Upregulated	0.00580	0.906	0.00136
**hsa‐miR‐21‐5p**	hsa‐mir‐21	Upregulated	0.00020	1.138	0.00008
**hsa‐miR‐223‐3p**	hsa‐mir‐223	Upregulated	0.00516	0.949	0.00197
hsa‐miR‐424‐5p	hsa‐mir‐424	Upregulated	0.02595	−0.10022	0.09163
hsa‐miR‐375	hsa‐mir‐375	Downregulated	0.00027	−1.82663	0.13509
hsa‐miR‐205‐5p	hsa‐mir‐205	Downregulated	0.00131	1.47158	0.41074
hsa‐miR‐143‐3p	hsa‐mir‐143	Downregulated	0.00280	−1.72661	0.10537
hsa‐miR‐203a‐3p	hsa‐mir‐203a	Downregulated	0.00699	−1.09907	0.60401
hsa‐miR‐192‐5p	hsa‐mir‐192	Downregulated	0.01563	0.27979	0.20536
hsa‐miR‐27b‐3p	hsa‐mir‐27b	Downregulated	0.02094	−0.79117	0.15092
hsa‐miR‐194‐5p	hsa‐mir‐194‐1	Downregulated	0.03672	0.07901	0.29485
hsa‐let‐7c	hsa‐let‐7c	Downregulated	0.04733	−1.24124	0.13345

miRNA expression in this article is tested with the novel RRA method. miRNA expression in TCGA is tested with paired *t*‐test (two‐sided). The miRNA with bold text is coincident and significant between published article data and TCGA data.

### Predicting, integrating, and verifying target genes

To further explore the function and mechanism of these five miRNAs in esophageal cancer, we identified the predicted target genes for each miRNA in six databases with the given integration strategy. The expression levels of these target genes were analyzed in 13 paired esophageal cancer and adjacent noncancerous tissue samples. In total, 1969 target genes were predicted and 686 of these target genes are verified as having significantly different expression between cancer and non‐cancer tissues (*P* ≤ 0.05). Then, 384 target genes were confirmed with a further filter (hsa‐miR‐100‐5p and hsa‐miR‐133b: log_2_ [fold change] >0; hsa‐miR‐155‐5p, hsa‐miR‐21‐5p, and hsa‐miR‐223‐3p: log_2_ [fold change] <0) (Table 3).

**Table 3:** Significant miRNA target genes in TCGA

miRNA	Significant target genes (*P* ≤ 0.05 and the log_2_ [fold change])
miR‐100‐5p	*CDK6, EIF5A, PSMA5, BAZ1B, LSM5, ATP1B3, RNFT1, CLCN5, COL4A1, RAB15, NKX3‐1, RB1, DDX21, EWSR1, YWHAE, NMT1, TTC30A, LIN28B, AP1AR, ABCB6, GNA13, GRB2, WDR4, ORC5, CCZ1B, CTDSPL2*
miR‐133b	*SMARCD1, MET, ZNF131, FOXC1, COL5A3, SLC39A1*
miR‐155‐5p	*CAB39L, METTL7A, INA, C3orf18, TTC37, PGRMC2, MITF, CD36, ATPAF1, PCDH9, MUT, TBC1D14, GABARAPL1, PDCD4, KIAA0430, KRCC1, ANAPC16, SETMAR, CDH2, EEF1A2, ZNF652, ERGIC1, RAB11FIP2, SAP30L, ZIC3, PHF17, GPT2, AGTR1, SKIV2L2, GHITM, DMD, ATP6V1H, PNPLA4, STIM1, NOVA1, TAB 2, JUN, SACM1L, CBR4, PCYOX1, ZNF493, GPM6B, OXCT1, AKR1C3, PALLD, CFL2, CAT, HSD17B12, IPO8, LTN1, PAK7, RIOK2, EIF3L, MPP2, UAP1, CYP2U1, FLNA, KANK2, TMBIM6, PIK3R1, MRS2, FOXO3, FGF2, SLC9A3R2, MPP5, MARCH2, PACSIN2, PRKAR2A, ARL6IP5, OSBPL9, ALDH1A2, PEBP1, ALDH3A2, NSA2, SNAP29, AGL, DDX17, ECI1, FAR1, AP1G1, INTS6, CRAT, MAN1A2, GLIPR2, PSIP1, XPC, MLH1, NR3C1, WRB, TNKS1BP1, RBPJ, TMED7‐TICAM2, CLIC4, SMAD4, SOX1, CSNK1A1, C16orf62, UGDH, SPECC1, NCKAP1, LONP2, TSHZ3, ZNF561, TRAK1, CTNNA1, RAB27B, ADH5, RAB6C, CLGN, CHD9, MEF2A, SNTB2, PKIA, LARS*
miR‐21‐5p	*FOXN3, FBXL17, LONRF2, CPEB3, LIFR, EPM2A, PAIP2B, PGRMC2, TGFBR3, LYRM7, LIMCH1, PDCD4, SASH1, ECI2, GPD1L, FBXL5, ARHGAP24, PURA, NFIA, FMOD, DCAF8, BTG2, CCNG1, ACAT1, KLF9, RNF11, UTRN, NBEA, ZBTB47, RAB11FIP2, DOCK3, SLAIN2, PRKAB2, FBXO3, ELAVL4, C20orf194, ISCU, PPM1L, PHF17, NEK1, RECK, COBLL1, MOAP1, GNAQ, DMD, ZBTB20, PDGFD, DDR2, FKBP5, PBX1, BDH2, FNBP1, MYO9A, TACC1, TIMP3, USP47, CYBRD1, MEGF9, BOC, SACM1L, DYNC1LI2, ATRX, FILIP1L, GOLGA4, ZBTB8A, TPRG1L, PALLD, TCF21, MYEF2, ZNF667, SOD3, PPAP2A, RHOB, WNK3, MKNK2, RUFY3, CALD1, TMX4, PRICKLE2, WFS1, SERPINI1, TUBGCP5, PHACTR2, SLMAP, PIK3R1, ARID4A, FERMT2, FAM63B, RNF38, IPP, SATB1, PER3, WDR47, TGFBR2, WDR7, PIGN, KLHL3, TSC1, MPP5, MON2, SEC63, CDS2, ARHGEF12, REV3L, SYBU, APPL1, AGGF1, JMY, BTBD3, WNK1, SESN1, DAAM1, TMEM56, PRKCE, SERAC1, ZYG11B, APOLD1, SPG11, FIGN, ANKRD46, PTGFR, PKD2, TM9SF3, MPDZ, RXRA, PTEN, CEP104, ITSN2, EIF4EBP2, CLOCK, ATP2B4, DCAF10, HECTD1, EIF2AK3, ZADH2, CSNK1A1, ESYT2, OSR1, CAPN2, DOCK8, NR2C2, MYCBP2, TEK, PTX3, VCL, ZRANB1, CYP4V2, FAM46A, TSHZ3, GPR64, SREK1, NIN, TPM1, PPP1R3B, EIF4A2, ARIH2, RAB6C, MLXIP, MTMR12, ABCD3, PPARA, MEF2A, NEK7, TOPORS, LARS*
miR‐223‐3p	*SLC2A4, ERO1LB, NFIA, FBXO8, RHOB, NFIX, ZFHX3, FBXW7, EPB41L3*

The targeted genes participating in pathways (KEGG and Panther enrichment pathways) are tested in the TCGA database, and then screening with the rules for hsa‐miR‐100‐5p and hsa‐miR‐133b: *P* ≤ 0.05, and log_2_ (fold change) >0; hsa‐miR‐155‐5p, hsa‐miR‐21‐5p and hsa‐miR‐223‐3p: *P* ≤ 0.05, and log_2_ (fold change) <0.

### Functional enrichment of target genes for miRNA

Overall, 38 KEGG pathways (Kyoto Encyclopedia of Genes and Genomes), and 22 Panther pathways were enriched for the target genes of hsa‐miR‐100‐5p, hsa‐miR‐133b, hsa‐miR‐155‐5p, hsa‐miR‐21‐5p, and hsa‐miR‐223‐3p.

In the KEGG pathways (Table 4), the target genes for hsa‐miR‐100‐5p were involved in cell growth and death, the immune system, the digestive system, and various cancers. Hsa‐miR‐133b functions as a signaling molecule and in cell‐cell interaction, cell communication, the digestive system, and cancer. Hsa‐miR‐155‐5p functions in cell communication, cell motility, transport and catabolism, signal transduction, cancer, and the immune system. Hsa‐miR‐223 functions in cancer protein folding, sorting, and degradation.

**Table 4:** KEGG pathway of the target genes for these five miRNAs

miRNA	Classification	KEGG pathway	Hyp‐c	Gene in pathway
miR‐155‐5p	Cell communication	Focal adhesion	0.036	*FLNA, PIK3R1, JUN, PAK7*
	Cell motility	Regulation of actin cytoskeleton	0.016	*FGF2, CFL2, NCKAP1, PIK3R1, PAK7*
	Transport and catabolism	Peroxisome	0.023	*CAT, CRAT, FAR1*
	Signal transduction	ErbB signaling pathway	0.030	*PIK3R1, JUN, PAK7*
	Cancers	Pathways in cancer	0.004	*FGF2, CTNNA1, PIK3R1, JUN, MITF, SMAD4, MLH1*
	Immune system	T‐cell receptor signaling pathway	0.036	*PIK3R1, JUN, PAK7*
		Toll‐like receptor signaling pathway	0.037	*PIK3R1, JUN, TAB 2*
miR‐21‐5p	Cell communication	Focal adhesion	0.011	*CAPN2, PDGFD, PIK3R1, PTEN, VCL*
		Tight junction	0.010	*MPP5, PTEN, MPDZ, PRKCE*
	Cell growth and death	p53 pathway feedback loops 2	0.005	*PIK3R1, PTEN, CCNG1*
		p53 signaling pathway	0.009	*SESN1, PTEN, CCNG1*
	Signal transduction	Phosphatidylinositol signaling system	0.012	*PIK3R1, CDS2, PTEN*
		Wnt signaling pathway	0.041	*PRICKLE2, CSNK1A1, DAAM1*
	Folding, sorting, and degradation	Protein processing in endoplasmic reticulum	0.012	*CAPN2, EIF2AK3, SEC63, WFS1*
	Cell growth	Angiogenesis	0.006	*TEK, PDGFD, RHOB, PIK3R1, PRKCE*
	Cancers	Pathways in cancer	0.025	*RXRA, APPL1, TGFBR2, PIK3R1, PTEN*
	Endocrine system	Adipocytokine signaling pathway	0.009	*RXRA, PRKAB2, PPARA*
		Insulin signaling pathway	0.007	*TSC1, MKNK2, PRKAB2, PPP1R3B, PIK3R1*
		Insulin pathway‐protein kinase B signaling cascade	0.005	*TSC1, PIK3R1, PTEN*
	Immune system	Fc gamma R‐mediated phagocytosis	0.014	*PPAP2A, PIK3R1, PRKCE*
miR‐223‐3p	Folding, sorting, and degradation	Protein processing in endoplasmic reticulum	0.041	*ERO1LB*
		Ubiquitin‐mediated proteolysis	0.042	*FBXW7*
miR‐100‐5p	Cell growth and death	Cell cycle	0.000	*CDK6, ANAPC1, YWHAE, ORC2, ORC5, PLK1, RB1*
		Oocyte meiosis	0.017	*ANAPC1, YWHAE, PLK1*
	Replication and repair	Base excision repair	0.018	*APEX1, HMGB1*
	Cancer	Pathways in cancer	0.011	*CDK6, NKX3‐1, GRB2, COL4A1, RB1*
	Digestive system	Protein digestion and absorption	0.012	*ACE2, COL4A1, ATP1B3*
	Immune system	Chemokine signaling pathway	0.012	*CCL7, GRB2, CXCL16, CXCL13*
miR‐133b	Cell communication	Adherens junction	0.025	*MET*
		Focal adhesion	0.007	*MET, COL5A3*
	Signaling molecules and interaction	Cytokine‐cytokine receptor interaction	0.048	*MET*
		ECM‐receptor interaction	0.023	*COL5A3*
	Cancers	Melanoma	0.029	*MET*
		Renal cell carcinoma	0.029	*MET*
	Digestive system	Protein digestion and absorption	0.024	*COL5A3*

In the Panther pathway analysis (Table 5), the target genes for hsa‐miR‐133b were enriched in signal transduction. Hsa‐miR‐155‐5p mainly functions in cancer development and signal transduction. Hsa‐miR‐21‐5p mainly functions in cell growth and death, cancer development, the endocrine system, and signal transduction. Hsa‐miR‐223‐3p functions in cell growth and death, cancer development, and signal transduction. However, no Panther pathway was enriched for target genes of hsa‐miR‐100‐5p.

**Table 5:** Panther pathway of the target genes for hsa‐miR‐100‐5p, hsa‐miR‐133b, hsa‐miR‐155‐5p, hsa‐miR‐21‐5p, and hsa‐miR‐223‐3p

miRNA	Classification	Panther pathway	Hyp‐c	Target genes in pathway
miR‐133b	Signal transduction	Wnt signaling pathway	0.048	*SMARCD1*
miR‐155‐5p	Development	Angiogenesis	0.048	*PIK3R1, FGF2, RBPJ, JUN*
	Signal transduction	Hedgehog signaling pathway	0.033	*PRKAR2A, CSNK1A1*
		Wnt signaling pathway	0.035	*SMAD4, CDH2, CSNK1A1, PCDH9, CTNNA1*
miR‐21‐5p	Cell growth and death	p53 pathway	0.028	*CCNG1, PIK3R1, PTEN*
		p53 pathway by glucose deprivation	0.023	*TSC1, PRKAB2*
		p53 pathway feedback loops 2	0.015	*CCNG1, PIK3R1, PTEN*
		PDGF signaling pathway	0.023	*PIK3R1, MKNK2, RHOB, NIN*
	Development	Angiogenesis	0.012	*PIK3R1, PRKCE, PDGFD, RHOB, TEK*
		Endothelin signaling pathway	0.025	*PIK3R1, PRKCE, GNAQ*
	Endocrine system	Insulin/IGF pathway‐protein kinase B signaling cascade	0.021	*PIK3R1, TSC1, PTEN*
	Signal transduction	Hypoxia response via HIF activation	0.027	*PIK3R1, PTEN*
		PI3 kinase pathway	0.015	*PIK3R1, GNAQ, PTEN*
miR‐223‐3p	Cell growth and death	PDGF signaling pathway	0.047	*RHOB*
	Development	Angiogenesis	0.046	*RHOB*
		Axon guidance mediated by Slit/Robo	0.028	*RHOB*
		Cytoskeletal regulation by Rho GTPase	0.032	*RHOB*
		Ras Pathway	0.042	*RHOB*
	Signal transduction	Integrin signaling pathway	0.041	*RHOB*
		Notch signaling pathway	0.033	*FBXW7*

No Panther pathway is enriched for target genes of hsa‐miR‐100‐5p.

### Effects of differentially expressed miRNAs on prognosis

**Figure 2:**
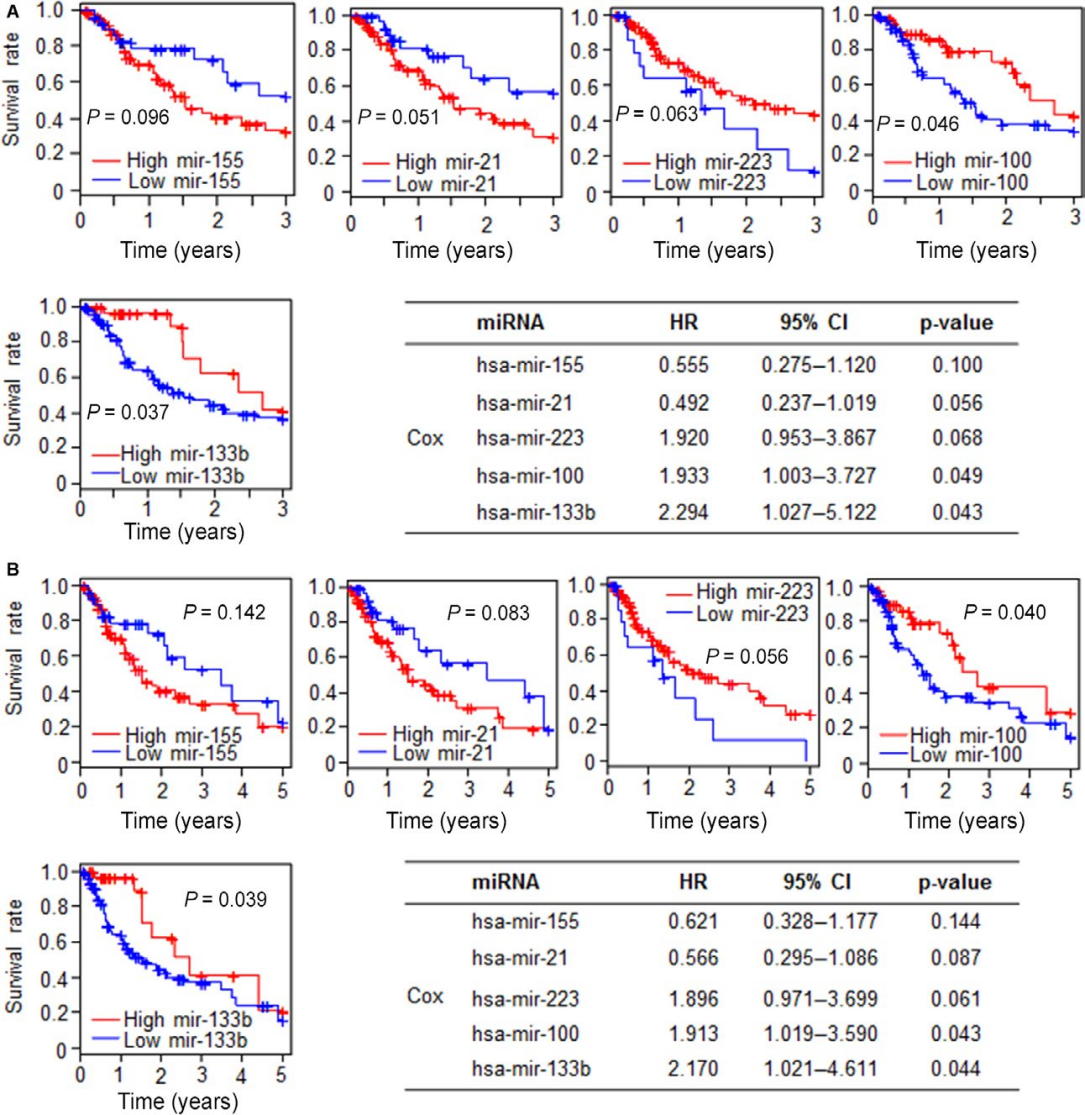
Prognostic value of miRNA in esophageal cancer. The survival rate analysis and Cox regression analysis are applied to hsa‐miR‐100‐5p, hsa‐miR‐133b, hsa‐miR‐155‐5p, hsa‐miR‐21‐5p, and hsa‐miR‐223‐3p for determination of prognosis in esophageal cancer. Here, the P value for survival analysis describes the significance of the log‐rank. (A) 3‐year‐survival rate analysis and 3‐year‐Cox regression analysis. (B) 5‐year‐survival rate analysis and 5‐year‐Cox regression analysis. HR, hazard ratio; CI, confidence interval.

To evaluate the prognostic impact of these five candidate miRNAs, survival and Cox regression analyses were applied to determine the risk of death and disease progression related to miRNAs in 185 esophageal cancer patient samples (Table 6). Owing to the complexity of miRNA metabolism, we selected the stem‐loop of mature miRNAs to standardize the analysis. The survival analysis results (Fig. 2) show that hsa‐mir‐100 and hsa‐mir‐133b were significantly associated with 3‐year and 5‐year survival, respectively. However, the other three miRNAs showed no significant difference in both 3‐year and 5‐year survival. Additionally, high hsa‐mir‐223 level was associated with a better prognosis in cancer, in contrast to the other miRNAs.

**Table 6:** Characteristics of the 185 esophageal cancer patients in TCGA

Characteristics	Frequency (No.)
Age	
<40	1.6% (3)
40–49	11.9% (20)
50–59	30.3% (56)
60–69	18.9% (35)
70+	31.4% (58)
Unknown	7.0% (13)
Sex	
Male	80.5% (149)
Female	12.4% (23)
Unknown	7.0% (13)
Alcohol history	
No	27.0% (50)
Yes	54.1% (100)
Unknown	8.1% (15)
Smoking history	
Nonsmoker	27.0% (50)
Current smoker	19.5% (36)
≤15 years	18.9% (35)
>15 years	18.4% (34)
Unknown	16.2% (30)
Neoplasm histologic grade	
G1	9.7% (18)
G2	38.9% (72)
G3	23.2% (43)
GX	21.1% (39)
Unknown	7.0% (13)
Clinical stage	
Stage I	1.6% (3)
Stage II	14.6% (27)
Stage III	11.4% (21)
Stage IV	5.4% (10)
Unknown	67.0% (124)
Radiation therapy	
No treatment	66.5% (123)
Treatment	16.8% (31)
Unknown	16.8% (31)
Treatment prior to surgery	
No treatment	26.5% (49)
Radiation and chemotherapy	1.1% (2)
Unknown	74.6% (138)
Additional pharmaceutical therapy	
No	3.2% (6)
Yes	5.4% (10)
Unknown	91.4% (169)

## Discussion

In general, the individual risk grade and decision of treatment largely depend on pathological and clinical factors, which show great variation between individuals, thereby influencing the predictive accuracy in cancer. A recent study demonstrates that differential expression of miRNAs can reflect tissue‐specific expression signatures through promotion or suppression of tumor development and progression 38. The application of miRNA‐based biomarkers for diagnosis thus provides a promising alternative.

In our study, we identified five differentially expressed miRNAs in esophageal cancer with comprehensive analyses of reported miRNA‐microarray sequencing data, miRNA‐generated sequencing data from the TCGA database, and RT‐PCR data from published studies. Here, we used paired cancer and adjacent noncancerous tissue samples to test miRNA and gene expression, which is more representative of the physiological status in the body than cell or serum samples. These five miRNAs are mainly involving in regulating esophageal cancer occurrence and development, according to comprehensive considerations of KEGG and Panther enrichment analyses. Additionally, high expressions of hsa‐mir‐100 and hsa‐mir‐133b, and low expressions of hsa‐mir‐155 and hsa‐mir‐21 tended to show a better prognosis, especially for hsa‐mir‐100 and hsa‐mir‐133b, which suggested high clinical value for esophageal cancer. Notably, hsa‐mir‐223 levels were increased in cancer tissue compared to normal tissue, but a good prognosis was also associated with a high expression level. In our results, hsa‐miR‐223‐3p mainly participates in protein folding, sorting, and degradation (Tables 4 and 5), which may underlie the anti‐tumor effects. Some studies have regarded the expression levels of different miRNAs as prognostic biomarkers in various cancers such as lung cancer 39, gastric cancer 40, and colorectal cancer 41. In our work, hsa‐miR‐100‐5p, hsa‐miR‐133b, hsa‐miR‐155‐5p, and hsa‐miR‐21‐5p were identified as differentially regulated, and associated with good prognosis; therefore, they can be used as miRNA biomarkers to increase the predictive ability in esophageal cancer, which will provide more choices in the treatment of esophageal cancer with further study. Hsa‐miR‐223‐3p also showed different expression in esophageal cancer tissue. Two or more of these miRNAs as testing indices could further improve the diagnostic accuracy of esophageal cancer.

Some significant target genes in our analysis have been verified experimentally in previous studies, for example, *SMARCD* 42 and *PTEN* 43, which play important roles in esophageal cancer occurrence and development (Table 5). Study of the other genes in Tables 4 and 5 may provide more constructive suggestions for esophageal cancer prognosis and treatment.

## Conflict of Interest

The authors declare no conflicts of interest.
