# Supplementary figures and images for: Clinical value of integrated‐signature miRNAs in esophageal cancer
Source: Cancer Med. 2017 Jul 14;6(8):1893–903. doi: 10.1002/cam4.1129 (PMC5548877; doi:10.1002/cam4.1129)

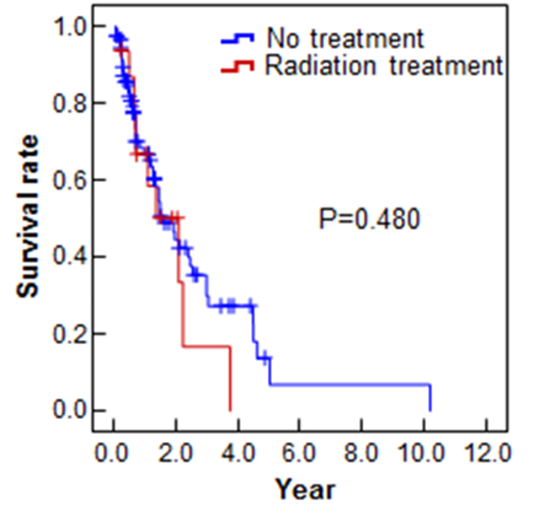

Supplement: Supplementary file 1 [file CAM4-6-1893-s001.tif]
